# Beyond resorption: targeting osteoclast fusion and polarization to restore balanced bone remodeling

**DOI:** 10.3389/fphar.2026.1821755

**Published:** 2026-06-05

**Authors:** Shunsuke Uehara, Midori Nakamura, Yasuhiro Kobayashi, Nobuyuki Udagawa

**Affiliations:** 1 Department of Biochemistry, Matsumoto Dental University, Nagano, Japan; 2 Institute for Oral Science, Matsumoto Dental University, Nagano, Japan

**Keywords:** anti-bone resorption, coupling, osteoclast fusion, osteoclast polarization, osteoporosis

## Abstract

The prevalence of osteoporosis is increasing worldwide as populations age, creating a growing clinical burden of fragility fractures and highlighting limitations of current antiresorptive therapies. Conventional agents such as bisphosphonates and denosumab effectively reduce fracture risk but suppress osteoclast number and activity indiscriminately, potentially impairing bone remodeling dynamics and silencing osteoclast-derived anabolic and angiogenic coupling signals. Recent advances have redefined osteoclasts as multifunctional cells that not only resorb bone but also orchestrate osteoblast differentiation and type H angiogenesis through factors such as PDGF-BB, S1P, Wnt10b, BMP6, and CTHRC1. These insights underscore the need for therapeutic strategies that temper pathological resorption while preserving beneficial coupling. This review integrates emerging molecular mechanisms regulating two key functions of osteoclasts, progenitor cell fusion and functional polarization, and evaluates their translational potential as selective antiresorptive targets. Fusion is driven by fusogen (DC-STAMP, OC-STAMP, Atp6v0d2, CD9, integrins), recognition systems (DC-STAMP, Siglec-15-sialylated TLR2), and alterations in membrane-cortical adhesion mediated by phosphatidylserine exposure, annexin A5, ERM, and BAR proteins. Osteoclast polarization relies on integrin αvβ3–Src–Pyk2 signaling, Rho-family GTPases. Recently, leucine-rich repeat kinase (LRRK1) has attracted attention as a factor that integrates both c-Src signaling and Rho-family GTPase signaling. Therapeutically, multiple modalities such as neutralizing antibodies against DC-STAMP/OC-STAMP, Siglec-15 inhibitors, small molecules such as E8431 (DC-STAMP antagonist) and C21 (Dock5 inhibitor), and LRRK1 inhibitors demonstrate the feasibility of selectively modulating fusion or polarization while maintaining osteoblast-coupling pathways. These strategies may complement conventional antiresorptives to provide safer, more physiologically balanced osteoporosis treatments. Collectively, emerging evidence positions osteoclast fusion and polarization as highly selective and clinically promising targets. A future therapeutic framework may integrate: (i) modest suppression of osteoclast number, (ii) targeted fusion inhibition to preserve preosteoclast-derived blood vessel formation, and (iii) polarization-directed modulation to reduce resorption while sustaining bone formation.

## 1 Introduction

The transition to an ageing society is driving a sustained increase in the prevalence of osteoporosis and burden of fractures. Globally, approximately one in three women and one in five men aged 50 years or older will suffer an osteoporotic fragility fracture during their lifetime, significantly impacting morbidity, mortality, and healthcare costs (Morin et al., 2025). Health economic analyses reveal direct costs in the tens of billions of dollars per year in high-income countries alone, estimated at approximately $17.9 billion in the US and £4 billion in the UK, figures that were expected to increase as life expectancies in these countries increase (Clynes et al., 2020). Meta-analysis data suggest a pooled global prevalence of approximately 18%, with gender-specific estimates of approximately 23% in women and 12% in men. Regional variation is significant, with particularly high prevalence rates reported in some African cohorts, highlighting heterogeneity in risk environments and diagnostic access (Salari et al., 2021). In the oldest-old population, fragility fractures occur in combination with frailty, sarcopenia, and multimorbidity. While fall prevention and nutritional optimization remain central to care, pharmacological fracture prevention is underutilized worldwide (Aspray and Hill, 2019; Clynes et al., 2020; Morin et al., 2025). Bone mass is maintained through a dynamic balance between bone resorption mediated by osteoclasts and bone formation mediated by osteoblasts. Accordingly, pharmacological treatments for osteoporosis are generally based on two therapeutic strategies: inhibition of bone resorption or stimulation of bone formation. In recent years, it has been emphasized that postmenopausal osteoporosis is not solely caused by hormonal imbalances, but is also significantly influenced by the interaction between immunity and bone (Fischer and Haffner-Luntzer, 2022; Qi et al., 2025; Wu et al., 2021). It has become clear that multiple immune cell populations, such as T cells, B cells, macrophages, neutrophils, and mast cells, regulate bone remodeling by controlling osteoclast formation and function through inflammatory cytokines such as TNFα, IL-6, IL-17, and histamine. Therefore, intensive research is being conducted on strategies for treating osteoporosis, repairing fractures, and regenerating bone by controlling these intercellular interactions, including the production of these cytokines. There is value in investigating therapeutic strategies that target osteoclasts as the convergence point of these intercellular interactions. In this review, we summarize the current status and challenges of therapeutic approaches employing antiresorptive agents. We also introduce potential molecular targets that may serve as the basis for next-generation osteoporosis therapies.

## 2 Current status of osteoporosis treatment targeting bone resorption

### 2.1 Osteoclast

Osteoclasts, the primary bone-resorbing cells, differentiate from monocyte–/macrophage-lineage precursors under the control of multiple extracellular and intracellular regulators (Udagawa et al., 2021; Zhang S. et al., 2025). Central to this process is the receptor activator of nuclear factor-κB ligand (RANKL)–RANK–osteoprotegerin (OPG) axis. In this axis, osteoblast- and osteocyte-derived RANKL promotes osteoclastogenesis, while OPG acts as a decoy receptor that inhibits the formation of osteoclasts and the bone-resorbing activity of osteoclasts. High-resolution single-cell analyses revealed a stepwise differentiation trajectory, including a transient CD11c^+^ precursor stage that was essential for RANK-mediated signaling, and identify Cited2 as a key transcriptional switch for terminal osteoclast commitment (Tsukasaki et al., 2020). Multiple transcription factors, including nuclear factor of activated T-cells cytoplasmic 1 (NFATc1), AP-1, microphthalmia-associated transcription factor (MITF), and PU.1, integrate RANKL signals to activate the osteoclastogenic program (Astleford et al., 2020). Zhang et al. showed that NF-κB promotes differentiation by suppressing miR-1276 expression and upregulating MITF (Zhang S. et al., 2020).

Monocyte–macrophage lineage cells differentiate into mononuclear osteoclast precursors (preOCs) in response to two essential cytokines, RANKL and macrophage colony-stimulating factor (M-CSF) (Boyle et al., 2003; Teitelbaum, 2000). Subsequent cell–cell fusion of these preOCs gives rise to multinucleated osteoclasts. Mature osteoclasts undergo polarization accompanied by cytoskeletal reorganization, leading to the formation of a ruffled border on their bone-facing surface (Väänänen et al., 2000). Through this ruffled border, osteoclasts secrete protons and proteolytic enzymes, including matrix metalloproteinase 9 (MMP9) and cathepsin K, to degrade the mineral and organic components of bone (Novack and Teitelbaum, 2008).

### 2.2 Limitations of existing antiresorptives: bisphosphonates and denosumab

Bisphosphonates remain the first-line treatment in many guidelines due to their efficacy, fracture risk reduction, oral and parenteral administration options, and cost-effectiveness. Their unique pharmacology, high affinity for bone mineral and selective uptake by osteoclasts during resorption, concentrates drug at remodeling surfaces (Drake et al., 2008). After endocytic internalization, the intracellular pharmacological actions of BPs vary depending on the R2 side chain structure (Billington and Reid., 2020). Nitrogen-free bisphosphonates (e.g., etidronate, clodronate) are metabolized to non-hydrolyzable ATP analogs, inhibiting ATP-dependent processes and inducing osteoclast apoptosis. In contrast, nitrogen-containing bisphosphonates (e.g., alendronate, risedronate, zoledronate) exert their primary effects via potent inhibition of farnesyl pyrophosphate synthase in the mevalonate pathway, thereby inhibiting the prenylation of small GTP-binding proteins, which are essential for cytoskeletal organization, vesicle trafficking, and cell survival. The accumulation of upstream metabolites, including isopentenyl pyrophosphate, further promotes osteoclast apoptosis via the formation of the cytotoxic ATP analog ApppI. There is concern that excessive suppression of bone metabolism due to long-term administration of BP preparations may result in significant inhibition of bone remodeling and rare atypical femoral fractures (Billington and Reid., 2020; Drake et al., 2008; Jelin-Uhlig et al., 2024). Other side effects include gastrointestinal intolerance with oral administration, an acute-phase response mediated by gamma delta T cell activation after intravenous administration, transient hypocalcemia (particularly in patients with vitamin D deficiency or renal dysfunction), and, although extremely rare, osteonecrosis of the jaw (ONJ). While the risk of ONJ in oncology regimens is orders of magnitude higher than that in osteoporosis treatment, it remains a clinical consideration, particularly in association with invasive dental procedures and poor oral hygiene. Similar maxillofacial complications occur with other antiresorptive drugs, such as the RANKL inhibitor denosumab, leading to the broader term antiresorptive drug-associated osteonecrosis of the jaw (ARONJ). Denosumab, a fully human anti-RANKL monoclonal antibody, offers potent, reversible suppression of osteoclastogenesis by neutralizing RANKL in the extracellular compartment; it is cleared systemically without skeletal embedding (Hanley et al., 2012). Denosumab reduces vertebral and hip fractures in high-risk patients (Morin et al., 2025). Yet its very reversibility confers a distinct vulnerability: abrupt discontinuation can trigger a “rebound” surge in bone turnover markers [(e.g., carboxy-terminal collagen crosslinks (CTX) and type I procollagen-N-propeptide (P1NP)] and rapid loss of gained bone mineral density (BMD) with increased risk of multiple vertebral fractures if not transitioned to an antiresorptive sequence (“exit strategy”) (Kumar et al., 2025). As with bisphosphonates, denosumab may precipitate hypocalcemia in predisposed patients; ONJ risk exists but is low at osteoporosis doses compared to oncology settings (Hanley et al., 2012).

### 2.3 Osteoclasts actively promote bone formation via coupling factors and the angiogenic factor

**FIGURE 1:**
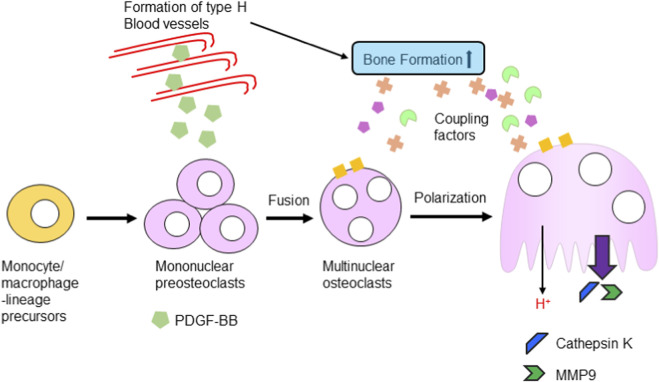
Differentiation process of osteoclasts and their roles in promoting bone formation. Mononuclear preOCs promote the formation of type H blood vessels through the secretion of PDGF-BB, which, in turn, enhances bone formation. Multinuclear osteoclasts enhance bone formation through the secretion of various coupling factors.

Osteoclasts are not simply cells that degrade bone; they secrete factors that (i) recruit and differentiate osteoprogenitor cells, (ii) promote the survival and function of osteoblasts, and (iii) induce the formation of specialized vasculature to support bone formation, thereby tightly coordinating resorption with subsequent formation (Figure 1). Sphingosine kinase 1 (SPHK1) expression is increased during osteoclast differentiation. Osteoclasts secrete sphingosine 1-phosphate (S1P). Intracellular S1P suppressed osteoclastogenesis (Ryu et al., 2006), whereas extracellular S1P promoted osteoblast precursor recruitment, survival, and mineralization (Pederson et al., 2008). In mice lacking the cathepsin K gene (Ctsk) specific to monocyte-osteoclast lineage cells, S1P levels were increased and bone formation was promoted via the S1P1/3 receptor on osteoblasts (Lotinun et al., 2013). This result suggests that S1P also contributes to the promotion of bone formation *in vivo*. Mature osteoclasts also express Wnt10b and bone morphogenetic protein (BMP) 6, both of which promote osteoblast formation and matrix mineralization. Inhibition of Wnt signaling with Dickkopf 1 suppressed osteoclast conditioned medium-induced mineralization. And the contribution of BMP6 was likewise functionally validated (Pederson et al., 2008). Independent studies have identified Wnt10b as a stimulator of postnatal osteoblast differentiation and bone formation. Transgenic mice expressing Wnt10b under the osteocalcin promoter showed increased BMD, whereas Wnt10b-deficient mice showed reduced bone formation rates (Bennett et al., 2007). Transforming growth factor-β1 released during bone resorption increased the expression of Wnt10b in osteoclasts through SMAD2/3 to enhance osteoblast mineralization (Ota et al., 2013). The collagen triple helix repeat containing-1 (CTHRC1) protein is both a positive regulator of osteoblastogenesis in osteoblast-lineage cells and, crucially, an osteoclast-secreted coupling factor induced by resorptive context (Kimura et al., 2008; Takeshita et al., 2013). Osteoclast-specific deletion of Cthrc1 caused osteopenia due to reduced bone formation, providing *in vivo* evidence that an osteoclast-produced molecule couples resorption to formation (Takeshita et al., 2013). Osteoclast-expressed ephrinB2 and osteoblast-expressed EphB4 engage in juxtacrine crosstalk; reverse signaling into preOCs suppressed osteoclastogenesis, while forward signaling into osteoblasts promoted osteogenic differentiation and increased bone mass (Zhao et al., 2006). In inflammatory bone defects, perturbing EphB4 reduced osteogenesis and promoted osteoclastic activity, emphasizing pathway relevance under pathological conditions (Shen et al., 2025). Collectively, these factors show that osteoclasts are indispensable cells for osteoblast recruitment and differentiation, tightly governing the amount and location of bone formation that succeeds resorption.

Angiogenesis is fundamental to bone modeling and remodeling. Specialized CD31^hi^Emcn^hi^ “type-H” blood vessels, abundant near growth plates and endosteal surfaces, create microenvironments that sustain osteoprogenitors and couple vascular growth to osteogenesis; these vessels decline with ageing, contributing to diminished formation (Kusumbe et al., 2014). In mice in which Notch signaling-induced gene expression was disrupted by knockout (KO) of the Rbpj gene specifically in vascular endothelial cells, not only the formation of type H blood vessels but also bone formation was suppressed (Ramasamy et al., 2014). PreOCs are central to this vascular–skeletal dialogue through platelet derived growth factor (PDGF)-BB. PDGF-BB from mononuclear TRAP + preOCs stimulates type H vessel formation and bone formation. In ovariectomized (OVX) mice, a model of postmenopausal osteoporosis, PDGF-BB and type H vessels were reduced, whereas administering PDGF-BB or increasing the pre-osteoclast pool by inhibiting cathepsin K restored type H vessel formation and improved bone mass in the OVX mice (Xie et al., 2014). Therapeutically, augmenting PDGF-BB in local contexts (e.g., fibrin sealants) improved angiogenesis and bone regeneration in experimental BRONJ models (Gao et al., 2021), and modulation of preOC biology (e.g., Siglec 15 neutralization) increased PDGF-BB secretion, enhanced bone formation, and accelerated fracture repair while simultaneously suppressed bone resorption (Zhen et al., 2021). In periodontal and alveolar bone, cathepsin K inhibition increased PDGF-BB and type H vessels, attenuating resorption in inflammatory settings (Zhou et al., 2024).

### 2.4 Cathepsin K inhibition: a promising but challenging therapeutic strategy

Cathepsin K, a cysteine protease highly expressed in osteoclasts, plays a central role in degrading type I collagen, the major organic component of bone matrix, during bone resorption. Given its osteoclast-specific expression and critical function, cathepsin K emerged as an attractive therapeutic target for selectively inhibiting bone resorption while preserving osteoclast viability and coupling activity. Odanacatib, a potent and selective cathepsin K inhibitor, demonstrated promising effects in early clinical trials, including significant reductions in bone resorption markers and increases in bone mineral density (Li et al., 2024). Importantly, because odanacatib does not induce osteoclast apoptosis, it was hypothesized that osteoclast-derived coupling factors would remain intact, potentially allowing continued bone formation despite reduced resorption. However, despite encouraging efficacy data, the development of odanacatib was halted after phase III trials due to an increased risk of serious adverse events, including stroke (McClung et al., 2019). This outcome highlighted the challenges of targeting osteoclast-specific pathways and underscored the need for safer therapeutic strategies.

### 2.5 Clinical implications

Present antiresorptives achieve fracture reduction largely by diminishing the number and the activity of osteoclasts. However, indiscriminate and sustained osteoclast suppression can blunt the remodeling dynamics and suppress osteoclast-derived anabolic and angiogenic signals necessary to maintain optimal bone quality. Therefore, there is a need for a treatment that suppresses pathological bone resorption while maintaining or promoting osteoclast-mediated coupling to bone formation.

## 3 Osteoclast fusion

### 3.1 Summary of osteoclast fusion

Fusion of mononuclear pre osteoclasts (preOCs) into multinucleated osteoclasts amplifies bone-resorptive capacity and determines cell size, sealing zone architecture, and pit formation efficiency (Søe, 2020). Selectively restricting fusion reduces bone resorption while preserving or enhancing secretion of PDGF BB, offering a promising therapeutic concept for conditions characterized by excessive bone loss (Cai et al., 2025; Xie et al., 2014; Zhen et al., 2021). The fusion process progresses through sequential steps, i.e., attraction/migration, recognition, adhesion, and membrane fusion, and depends on fusogenic molecules such as dendritic cell-specific transmembrane protein (DC-STAMP) and osteoclast stimulatory transmembrane protein (OC-STAMP), as well as structures such as zipper-like structures, tunneling nanotubes and invadosomes (Chernomordik and Melikov, 2025; Takito and Nakamura, 2020). Recent studies show that altered tension of plasma membrane driven by collapsed membrane–cortex attachment (MCA) is a crucial biophysical determinant of fusion, opening a new Frontier in osteoclast fusion biology (Dufrançais et al., 2025; Wan et al., 2025; Leikina et al., 2026).

### 3.2 Transcriptional control and core fusogens: NFATc1, DC-STAMP, OC-STAMP

RANKL is a cytokine essential for osteoclastogenesis. During RANKL-induced osteoclast differentiation, the transcription factor NFATc1 binds to the promoters of both DC-STAMP and v-ATPase V0 subunit d2 (Atp6v0d2) and upregulates their expression. Inhibition of NFATc1 activity in osteoclasts by cyclosporine treatment suppressed osteoclast fusion, whereas overexpression of both DC-STAMP and Atp6v0d2 restored multinucleation (Kim et al., 2008). Lee et al. reported that osteoclast fusion was selectively inhibited and bone resorption was suppressed in Atp6v0d2-deficient mice (Lee et al., 2006). These results suggest that RANKL-NFATc1 signaling promotes osteoclast fusion by upregulating the expression of factors required for osteoclast fusion.

#### 3.2.1 DC-STAMP

**FIGURE 2:**
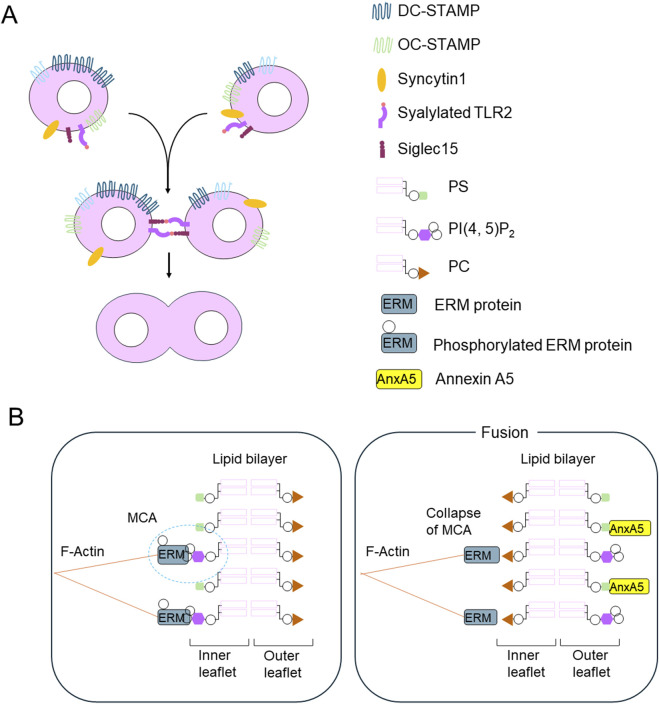
Mechanisms of osteoclast fusion. **(A)** DC-STAMP-high expressing mononuclear osteoclasts fuse with DC-STAMP-low expressing mononuclear osteoclasts, and Siglec15-syalylated TLR2 is involved in the recognition of fusion partners. **(B)** Exposure of negatively charged phospholipids, such as PS and PI(4, 5)P_2_, on the cell membrane surface and disruption of membrane-cortex attachment (MCA) promote osteoclast fusion. PS, phosphatidylserine; PC, phosphatidylcholine; PI(4, 5)P_2,_ phosphatidylinositol-4, 5-diphosphate.

Expression of DC-STAMP, which is essential for osteoclast fusion, was rapidly induced by RANKL. Knockdown by siRNA and DC-STAMP-specific neutralizing antibodies inhibited osteoclast fusion *in vitro* (Kukita et al., 2004). Yagi et al. generated DC-STAMP-deficient mice and reported that although the expression of osteoclast markers is normal, osteoclast multinucleation is completely inhibited in homozygotes. DC-STAMP-deficient mice exhibited osteopetrosis, which was due to the impaired multinucleation that reduced the bone-resorbing activity of osteoclasts (Yagi et al., 2005). RANKL stimulation induced differentiation into heterogeneous DC-STAMP-low expressing cells (DC-STAMP^lo^) and DC-STAMP-high expressing cells (DC-STAMP^hi^). DC-STAMP^lo^ cells acted as “master fusion factors,” while DC-STAMP^hi^ cells acted as mononuclear donors. These results highlight the asymmetric role of DC in fusion partnerships (Mensah et al., 2010) (Figure 2A). Studies using human tumor giant cells showed that DC-STAMP was phosphorylated on its tyrosine residues and physically interacted with SHP-1, an SH2 domain-containing tyrosine phosphatase, and CD16, an ITAM-associated protein. Studies using human tumor giant cells demonstrated that DC-STAMP contains a tyrosine-based inhibitory motif (ITIM) at its cytoplasmic tail, and that phosphorylation of the tyrosine residues in the ITIM is important for its interaction with Src homology region 2 domain-containing phosphatase-1 (SHP-1), an SH2 domain-containing tyrosine phosphatase and the immunoreceptor tyrosine-based activation motif (ITAM)-associated protein CD16. (Chiu et al., 2012). Additionally, studies showed that DC-STAMP supports the exposure of non-apoptotic phosphatidylserine (PS) in osteoclasts, which regulates the activity of syncytin-1 (Søe et al., 2011; Verma et al., 2018). These results suggest a crosstalk between lipid signaling and networks of protein fusion factors.

#### 3.2.2 OC-STAMP

OC-STAMP is a RANKL-induced, multi-pass transmembrane protein required for efficient fusion; knockdown/antibody blockade suppressed multinucleation, while overexpression augmented osteoclastogenesis (Yang et al., 2008; Witwicka et al., 2015). Although OC-STAMP KO mice had near normal basal bone mass, OC-STAMP proved pathogenic in inflammatory settings: in periodontitis, OC-STAMP upregulated CD9 (a tetraspanin permissive fusogen), and anti OC-STAMP antibody suppressed osteoclast fusion, pit formation, and alveolar bone loss (Ishii et al., 2018). Recent mouse genetics showed cooperativity: DC-STAMP and OC-STAMP act nonredundantly; fusion fails when both are absent or when complementary expression is segregated into separate cells (Homma et al., 2025).

### 3.3 Recognition and signaling, siglec-15- TLR2, DAP12/Syk, and metabolic inputs

#### 3.3.1 Siglec-15 and the DAP12/Syk hub

Sialic acid-binding immunoglobulin-type lectin (Siglec)-15 has emerged as a critical regulator of osteoclast differentiation and multinucleation. Initial transcriptomic analyses identified Siglec 15 as markedly upregulated in giant cell tumor of bone and during RANKL induced osteoclastogenesis in mouse and human precursor cells, suggesting a role in late-stage differentiation (Hiruma et al., 2011). Ishida-Kitagawa et al. revealed that Siglec 15 is an NFATc1-inducible gene that integrates RANKL signaling with ITAM pathways via its association with the adaptor protein, DNAX-activating protein of 12 kD (DAP12) (Ishida-Kitagawa et al., 2012). This Siglec 15–DAP12–Syk axis is required for cytoskeletal organization, actin-ring formation, and the development of functional multinucleated osteoclasts. Siglec 15 deficiency resulted in impaired osteoclast fusion and reduced bone-resorptive activity of osteoclasts both *in vitro* and *in vivo*, leading to mild osteopetrosis in mice (Hiruma et al., 2013). Siglec 15 deficiency resulted in impaired osteoclast fusion and reduced bone-resorptive activity of osteoclasts both *in vitro* and *in vivo*, leading to mild osteopetrosis in mice (Hiruma et al., 2013). Furthermore, loss of Siglec 15 attenuated RANKL-induced activation of PI3K/Akt and ERK signaling, pathways indispensable for osteoclast maturation (Kameda et al., 2013). Together, these findings establish Siglec 15 as a pivotal modulator of osteoclast multinucleation, coupling glycan recognition with ITAM-dependent signaling to orchestrate the formation of bone-resorbing osteoclasts. Mechanistically, Siglec 15 recognized sialylated Toll-like receptor 2 (TLR2) to mediate pre-fusion cell recognition; M-CSF licensed Siglec 15 expression, while RANKL induced ST3Gal1 to sialylated TLR2, enabling binding and initiating fusion (Dou et al., 2022) (Figure 2A). TLR2 deficient mice showed increased TRAP + cells yet reduced multinucleation and diminished resorptive ultrastructure, mirroring the recognition-to-fusion defect (Muneyama et al., 2023).

#### 3.3.2 Metabolic control feeds the fusion program

Emerging evidence highlights pyruvate kinase M2 (PKM2) as a central metabolic and signaling regulator of osteoclastogenesis across diverse pathological microenvironments. PKM2, traditionally recognized as a key glycolytic enzyme, exerts non-metabolic functions that integrate inflammatory cues, hypoxic adaptation, and promote osteoclast differentiation and bone resorption (Tang et al., 2019; Zou et al., 2021). In inflammatory settings such as periodontitis, PKM2 expression was enhanced by LPS-induced glycolytic reprogramming, and nuclear translocation of dimeric PKM2 was facilitated which, in turn, downstream effectors including signal transducer and activator of transcription (STAT)3 and hypoxia-inducible factor 1α (HIF 1α) were activated (Li et al., 2025). Inhibition of PKM2 or glycolysis attenuated inflammatory osteoclast formation, while activators such as TEPP 46 inhibited nuclear PKM2 signaling and protected against alveolar bone loss *in vivo*. Recent studies demonstrated that modulation of PKM2 activity profoundly influences osteoclast formation: both genetic knockdown and pharmacological activation of PKM2 reduce osteoclastogenesis by suppressing osteoclast specific transcripts and impairing precursor cell fusion through downregulation of OC-STAMP and DC-STAMP (Cai et al., 2025). Taken together, these findings suggest that PKM2 may be regarded as a metabolic checkpoint that integrates hypoxic inflammatory signals to control osteoclast differentiation and function. Targeting PKM2 therefore represents a promising therapeutic strategy for pathological bone loss associated with osteoporosis, as well as periodontitis.

### 3.4 Membrane mechanics, lipid codes, and supracellular structures

#### 3.4.1 ERM proteins mediated MCA tuning set the mechanical threshold

Ezrin/radixin/moesin (ERM) proteins are involved in MCA. Phosphorylation of threonine residues in the C-terminal region of ERM proteins is required for MCA (McClatchey, 2014). Wan et al. demonstrated *in vitro* that the ERM protein, ezrin, is required for osteoclast fusion using Raw264.7 cells (Wan et al., 2025). They also showed several Bin/Amphiphysin/Rvs (BAR) domain containing proteins such as Baiap2l and Fnbp1 are involved in osteoclast fusion as effecters of ezrin. Knockdown of ezrin, Baiap2l, and Fnbp1 inhibited the formation of invadosomes, structures involved in cell fusion, in Raw264.7 cells. Dufrançais et al. demonstrated *in vitro* and *in vivo* that the ERM protein moesin is required for osteoclast fusion via forming of tunneling nanotubes (TNTs), using mouse monocytes immortalized by overexpression of HoxB8 and moesin KO mice (Dufrançais et al., 2025). Both studies demonstrated that reduced membrane tension due to decreased phosphorylation of ERM proteins is required for osteoclast fusion (Figure 2B).

#### 3.4.2 TNTs: highways for fusogen traffic and pairing

TNTs are actin-based intercellular bridges that facilitate targeted exchange of membrane, vesicles, and fusogenic proteins (e.g., DC-STAMP) between precursors and support pairing before fusion (Li et al., 2019; Pennanen et al., 2017; Takahashi et al., 2013; Zhang et al., 2021). RANKL stimulation increased the expression of M-Sec, a protein required for TNT formation, in osteoclast precursor cells, thereby inducing TNT formation. Inhibition of TNT formation suppressed osteoclast multinucleation (Takahashi et al., 2013). Li et al. reported that TNTs also mediate crosstalk between endothelial progenitor cells and Raw264.7 cells, a monocyte/macrophage cell line, altering osteoclast differentiation via macrophage migration inhibitory factor (MIF) signaling (Li et al., 2019). In experiments using monocytes immortalized by introducing a construct linking the Hoxb8 gene to the estrogen receptor (ER) gene, it was revealed that the expression of integrin α2β1 (encoded by ITGA2) was increased during osteoclast differentiation. Although ITGA2 deficiency increased the length of TNTs, reduced expression of DC-STAMP and CD9 suppressed cell fusion, suggesting that TNT abundance alone does not trigger cell fusion (Brockhaus et al., 2024).

#### 3.4.3 Zipper-like structures (ZLS) orchestrate secondary fusion

ZLS are transient actin superstructures at cell–cell interfaces that predict higher multinucleation and transition into podosome belts (Takito et al., 2012; 2015; 2017; Takito and Nakamura, 2012; Wang et al., 2018). ZLS display symmetric retrograde actin flow (Arp2/3-dependent) and are negatively regulated by actomyosin contractility, consistent with the low-tension prerequisite for fusion (Dufrançais et al., 2025; Takito et al., 2012; 2017; Wan et al., 2025). Active vitamin D3 promoted ZLS formation via Src/Rac1, thereby enhancing osteoclast activity (Wang et al., 2018).

#### 3.4.4 Phosphatidylserine (PS) and annexins weaken cortex attachment to favor fusion

Recent studies have established phosphatidylserine (PS) exposure as a central regulator of preOC fusion and osteoclastogenesis. Non-apoptotic PS externalization, controlled by RANKL induced caspase 8 activation and downstream cleavage of the scramblase Xkr8, functions as a “fuse-me” signal that promoted membrane remodeling and enabled multinucleated osteoclast formation (Krishnacoumar et al., 2024; Verma et al., 2018). This process is further facilitated by extracellular annexins, including Annexin A5, which stabilized surface PS in preOCs (Leikina et al., 2026) (Figure 2B). Ca^2+^ signaling promoted surface exposure of PS and phosphatidylinositol-4, 5-diphosphate [PIP(4, 5)P_2_] and contributed to actin detachment from the plasma membrane. A computational approach revealed that this collapse of MCA partially strengthens the tension in the cell membrane, leading to localized membrane protrusions. PS also provides docking sites for regulatory proteins such as the unconventional low–molecular weight form of La protein. ROS dependent trafficking of La protein to the cell surface enhanced osteoclast multinucleation and resorptive activity (Leikina et al., 2024; Whitlock et al., 2023). Kang et al. reported that PS interacts with receptors such as T cell immunoglobulin and mucin domain-containing protein 4 (TIM4), brain-specific angiogenesis inhibitor 1 (BAI1), and Stabilin-2 (STAB2), and orchestrates lipid transporter regulation during osteoclast differentiation (Kang et al., 2020). Utilizing these properties, PS-containing liposomes have been shown to suppress osteoclastogenesis, reprogram inflammatory macrophages, and prevent pathological bone loss in models of arthritis, glucocorticoid-induced osteoporosis, and systemic inflammation (Eskandarynasab et al., 2020; Ma et al., 2011; Wu et al., 2010). More recently, PS incorporated exosome mimetics have been engineered to target preOCs and deliver therapeutic cargos, such as CXCR3 antagonists, effectively mitigating bone loss in osteoporotic models (Kang et al., 2024). Collectively, these findings highlight PS-mediated signaling and PS based nanotechnologies as powerful modulators of osteoclast biology with significant therapeutic potential for bone resorptive disorders.

### 3.5 Accessory fusogens and checkpoints

CD9 (tetraspanin) is RANKL-induced, localizes to lipid rafts, and is essential for fusion; overexpression induces spontaneous fusion, whereas raft disruption blocks multinucleation (Ishii et al., 2006). In experiments using bone marrow macrophages derived from CD47-deficient mice, the formation of multinucleated osteoclasts was suppressed (Koskinen et al., 2013; Lundberg et al., 2007; Maile et al., 2011). In vivo experiments using CD47-deficient mice, some reports showed a decrease in osteoclast numbers (Koskinen et al., 2013; Lundberg et al., 2007), while other showed no decrease. (Maile et al., 2011). Mechanistically, CD47 is involved in osteoclast fusion by binding to signal-regulatory protein (SIRP)α (also called SHPS-1) (Koskinen et al., 2013; Lundberg et al., 2007; Maile et al., 2011). The endogenous retroviral protein syncytin 1 and its receptor ASCT2 are expressed during osteoclast differentiation, and inhibition of syncytin 1 reduces osteoclast multinucleation and TRAP activity (Søe et al., 2011). Verma et al. reported that the fusion activity of syncytin-1 is regulated by annexins and S100A, as well as the extracellular exposure of PS. (Verma et al., 2018). Additional modulators include PSTPIP2 (F-BAR protein) as a negative feedback regulator of CSF-1R signaling that suppresses fusion (Chitu et al., 2012) and MAGI1, which restrains fusion via RhoA/ROCK1, impacting subchondral bone in osteoarthritis (Zhang J. et al., 2025).

### 3.6 Emerging genetic and epigenetic controls

Msx2 in myeloid cells acts as a “brake removal” for fusion by stabilizing PU.1 against FBXW7-mediated degradation; its deficiency expanded preOCs, increased PDGF-BB secretion, and augmented angiogenic coupling, preserving bone mass suggesting the potential for a preOC-centric anabolic therapy (Ma et al., 2025). Epigenetically, Arid1a increased chromatin accessibility at the Siglec-15 promoter via Jun/Fos, promoting fusion; Arid1a KO restricted Siglec-15 expression, inhibited fusion, and protected against OVX-induced bone loss (Zhang et al., 2024). High-throughput screens identified Calcrl, Marco, and Ube3a as additional fusion determinants, with Tmem26 acting as a negative regulator (Cho et al., 2022).

### 3.7 Concerns about excessive PDGF-BB

In recent years, accumulated evidence has revealed that overproduction of PDGF-BB is involved in the pathogenicity of several diseases. Su et al., using an osteoarthritis (OA) model, demonstrated that abnormal overproduction of PDGF-BB promotes vascular and sensory nerve intrusion into articular cartilage, which is normally avascular, accelerating OA progression and worsening joint pain (Su et al., 2020). Beyond the joints, elevated serum PDGF-BB levels have been shown to induce osteogenic differentiation of vascular parietal cells, leading to pathological vascular remodeling such as atherosclerosis and cerebral vascular calcification (Santhanam et al., 2021; Wang et al., 2023). These findings raise important concerns regarding osteoporosis treatment strategies aimed at increasing the number of pOCs that highly express PDGF-BB by inhibiting osteoclast fusion. Overproduction of PDGF-BB may unintentionally cause vascular calcification, atherosclerosis, or cerebrovascular dysfunction depending on the patient’s underlying condition. Therefore, careful control of PDGF-BB levels is essential, and this treatment strategy may be contraindicated in certain patient populations. Therefore, it is beneficial to consider alternative approaches as complementary or safer strategies to minimize the systemic risks associated with PDGF-BB overproduction while regulating bone remodeling, such as suppressing osteoclast polarization rather than bone fusion.

## 4 Osteoclast polarization

### 4.1 Summary of osteoclast polarization

Mature osteoclasts undergo pronounced polarization on bone surfaces, reorganizing actin-rich podosomes into clusters, rings, and ultimately a peripheral sealing zone that encloses the resorption lacuna (Blangy et al., 2020; Kong et al., 2020; Georgess et al., 2014). Within this sealed compartment, polarized exocytosis of secretory lysosomes generates the ruffled border, a convoluted membrane domain enriched in V ATPase proton pumps and the ClC 7 chloride channel, which acidify the lacuna to dissolve mineral, while MMP9 and cathepsin K degrade organic matrix (Ng et al., 2019; Novack and Teitelbaum, 2008). These cytoskeletal and trafficking dynamics are orchestrated by integrin αvβ3–dependent adhesion, the Src–Pyk2 signaling complex, and Rho-family GTPases, which regulate actin polymerization, microtubule organization, vesicle movement, and membrane remodeling (Teitelbaum, 2011; Uehara et al., 2018). Leucine-rich repeat kinase (LRRK)1 has emerged as a key integrator of these pathways and a potential therapeutic target (Si et al., 2018; Xing et al., 2013; Zeng C et al., 2016).

### 4.2 Integrin αvβ3–Src–Pyk2 axis as the central adhesion machinery

Integrin αvβ3 is the primary adhesion receptor that orchestrates osteoclast polarization [Teitelbaum., 2011]. Engagement of αvβ3 recruits a canonical signaling complex containing c-Src, Syk, Dap12, Slp76, Vav3, and Rac, which drives actin assembly, podosome turnover, and cell spreading. Src plays a dual role as a scaffold and kinase: it supports early podosome assembly and phosphorylates cortactin to regulate podosome maturation (Luxenburg et al., 2006). Genetic ablation of Src resulted in severe osteopetrosis due to failure to form ruffled borders and functional sealing zones (Boyce et al., 1992; Soriano et al., 1991).

Pyk2 acts as a central adhesion-dependent scaffold. Autophosphorylation of Tyr402 enabled binding to Src, forming a Pyk2-Src-Cbl complex that promoted downstream regulatory tyrosine phosphorylation and cytoskeletal remodeling. (Kong et al., 2020; Lakkakorpi et al., 2003). Notably, Tyr402 phosphorylation not intrinsic kinase activity is essential for osteoclast spreading and sealing zone formation. Upstream pathways such as Notch2 enhanced Pyk2 activation (Jin et al., 2016), whereas dynamin reduced Pyk2 phosphorylation in a Src-dependent manner (Bruzzaniti et al., 2009). Together, Src and Pyk2 form the core of the osteoclast adhesion signaling module.

### 4.3 Rho-family GTPases as master regulators of osteoclast cytoskeletal dynamics

#### 4.3.1 Overview of RhoA, Rac, and Cdc42 in osteoclasts

**FIGURE 3:**
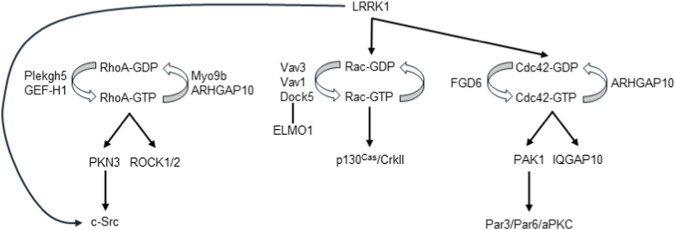
Signaling pathways of small GTPases in osteoclast polarization. The activities of various small GTPases and their effectors are regulated downstream of integrin-c-Src/Pyk2, RANKL-RANK, and M-CSF-c-Fms signals.

Rho-family small GTPases including RhoA, Rac1/2, and Cdc42 act as molecular switches that integrate signals from integrins, ITAM-associated receptors, chemokine receptors, and cytokines such as M-CSF and RANKL (Itzstein et al., 2011; Ross and Teitelbaum., 2005; Uehara et al., 2018). Their tightly regulated activity governs actin polymerization, microtubule stability, vesicle trafficking, and cell polarity. Disruption of these pathways consistently results in impaired sealing zone formation, defective ruffled border organization, and osteopetrosis, underscoring their centrality in osteoclast biology (Figure 3).

#### 4.3.2 RhoA signaling: PKN3, ROCK, and GEF/GAP regulation

RhoA activity must be precisely tuned: excessive activation destabilizes podosomes, whereas insufficient activity also impairs bone resorption. We reported that protein kinase N3 (PKN3), a RhoA effector was activated downstream of Wnt5a–Ror2–Daam2 signaling, which, in turn, enhances Src kinase activity and promotes sealing zone formation (Uehara et al., 2017). Loss of PKN3 or Ror2 resulted in impaired bone resorption and increased trabecular bone mass. ROCK1/2, the RhoA effectors, regulate actomyosin contractility, podosome patterning, and sealing zone stability (McMichael et al., 2014; Morel et al., 2024). Hyperactivation induced by microtubule depolymerization disrupted actin rings, whereas ROCK inhibition suppressed osteoclast-mediated bone degradation (Strzelecka-Kiliszek et al., 2017).

RhoA activity is further controlled by guanine nucleotide exchange factors (GEFs) and GTPase-activating proteins (GAPs). A growing body of evidence highlights the essential contribution of Rho-specific GEFs and GAPs to the organization of podosomes and the formation of the osteoclast sealing zone. Among these, Plekhg5 emerges as a differentiation-induced Rho-GEF that orchestrates cell polarity, migration, adhesion, and podosome patterning in both macrophages and osteoclasts (Iwatake et al., 2017). Loss of Plekhg5 disrupted podosome organization and bone resorption, accompanied by aberrant localization or expression of downstream Rho effectors such as EB1, cofilin, vinculin, mDia1, and LIMK1, underscoring its role in coordinating actin–microtubule crosstalk. Another key regulator is GEF-H1, a microtubule-associated RhoA-GEF whose activity was restrained by sequestration on polymerized microtubules (Morel et al., 2024). Microtubule depolymerization released GEF-H1, triggering RhoA–ROCK activation and consequent actin ring destabilization. Although partial depletion of GEF-H1 did not prevent sealing zone formation under steady-state conditions, it reduced sealing zone size and impaired resorption, indicating that finely tuned GEF-H1 activity is required for sealing zone stability. Complementing these GEFs, several Rho-GAPs also modulate osteoclast cytoskeletal dynamics. Myosin IXB (Myo9b), an actin-based motor protein with intrinsic RhoGAP activity, suppressed RhoA signaling to maintain proper podosome patterning, microtubule stability, and Src activation. Depletion of Myo9b resulted in elevated RhoA activity, disrupted podosome organization, and severely suppressed resorption (McMichael et al., 2014). Similarly, ARHGAP10, a GAP for Cdc42 and RhoA, functions as a microtubule-associated regulator whose BAR-PH domain mediates direct microtubule binding (Jentschel et al., 2025; Maurin et al., 2021; Steenblock et al., 2014). Loss of ARHGAP10 altered actin ring morphology and dynamics, leading to impaired resorptive activity, and rescue experiments demonstrated that both its microtubule-binding capacity and GAP activity are indispensable (Jentschel et al., 2025). Together, these findings reveal that osteoclast function relies on a precisely balanced network of Rho-GEFs and Rho-GAPs that integrate microtubule dynamics with Rho-GTPase signaling to control podosome organization and sealing zone integrity.

#### 4.3.3 Rac signaling: Vav proteins, and Dock5

Rac1/2 are indispensable for podosome assembly, sealing zone formation, and acidification of the resorption lacuna. Osteoclast-specific deletion of Rac1/2 resulted in severe osteopetrosis (Zhu et al., 2016). Vav3 is a major Rac GEF activated downstream of Syk following αvβ3 or M-CSF receptor engagement (Faccio et al., 2005). Vav3-deficient osteoclasts exhibited defective spreading and reduced resorption, and Vav3 KO mice were protected from RANKL- or PTH-induced bone loss. In contrast, Vav1 negatively regulates osteoclastogenesis, illustrating the fine balance of Rac activation (Jang et al., 2019). Dock5 is a regulator of the osteoclast cytoskeleton. It promoted Rac-dependent actin remodeling (Vives et al., 2011). In addition, Dock5 also promoted Rac-independent microtubule stabilization via Akt-mediated inhibition of GSK3β (Guimbal et al., 2019). Dock5 inhibition using with C21, a selective Dock5 inhibitor, disrupted podosome belts and protected against bone loss in models of osteoporosis, arthritis, and metastasis (Vives et al., 2015).

#### 4.3.4 Cdc42 signaling: polarity, vesicle trafficking, and osteoclastogenesis

Cdc42 is another essential Rho family GTPase that governs osteoclast polarity, vesicle trafficking, and differentiation. Osteoclast-specific deletion of Cdc42 resulted in osteopetrosis, impaired sealing zone formation, defective ruffled border organization, and resistance to OVX-induced bone loss (Ito et al., 2010). Cdc42 regulated M-CSF–dependent proliferation and survival by modulating cyclin D expression, Rb phosphorylation, and apoptosis pathways involving caspase-3 and Bim. It was also required for RANKL-induced activation of MITF and NFATc1, positioning Cdc42 as a key regulator of osteoclastogenesis. Mechanistically, Cdc42 participated in the Par3/Par6/aPKC polarity complex and coordinated actin dynamics through effectors such as IQGAP1 (Ito et al., 2010; Steenblock et al., 2014). Steenblock et al. reported that FGD6, a Cdc42 GEF, links integrin-mediated adhesion to podosome assembly and simultaneously regulates retromer-dependent membrane recycling via WASH on endosomes, thereby coupling cytoskeletal remodeling with ruffled border maintenance (Steenblock et al., 2014). Recently, it was suggested that TUBB6 may negatively regulate the binding of ARHGAP10, a Cdc42 GAP, to microtubules, thereby linking Cdc42 signaling with the regulation of microtubule dynamics. (Jentschel et al., 2025). These mechanisms further illustrate the integration of microtubule dynamics with local GTPase signaling.

#### 4.3.5 Additional regulators for Rho family GTPases

Beyond Rho, Rac and Cdc42, several additional pathways converge on the osteoclast cytoskeleton. Siglec-15, a DAP12-associated ITAM receptor, activated Rap1–Rac1 signaling through a complex containing p130Cas and CrkII; its deletion led to osteopetrosis and defective actin ring formation (Kobayashi et al., 2024). ELMO1, acting with DOCK proteins, promoted Rac activation and osteoclast differentiation, and its loss protected against bone erosion in inflammatory arthritis (Liang et al., 2021). CD55 supports osteoclast survival and resorptive function by enabling M-CSF–and RANKL-induced Rac activation. CD55 deficiency resulted in increased osteoclast numbers but impaired function and elevated bone mass (Shin et al., 2020). C-C chemokine receptor (CCR) 5 has been shown to be involved in centrosome clustering, microtubule formation, and cathepsin K secretion in osteoclasts. Loss of CCR5 disrupted osteoclast polarity, and the phenotype was rescued by constitutively activated Rac or Rho (Lee et al., 2023). Together, these factors highlight the extensive network of receptors, adaptors, and cytoskeletal regulators that converge on small GTPases to coordinate osteoclast adhesion, polarity, vesicle trafficking, and bone resorption.

### 4.4 LRRK1 as a central integrator of cytoskeletal and vesicular machinery

#### 4.4.1 Roles of LRRK1 in osteoclast function

Leucine-rich repeat kinase (LRRK)1 is a multidomain protein belonging to the ROCO family, containing ankyrin repeats, leucine-rich repeats, ROC GTPase-like domains, COR domains, kinase domains, and WD40 repeats (Zeng X et al., 2016). LRRK1 is indispensable for osteoclast function, whereas LRRK2 appears dispensable (Xing et al., 2013). Mice lacking LRRK1 exhibited severe osteopetrosis due to a profound defect in osteoclast-mediated bone resorption. LRRK1-deficient osteoclasts differentiated normally in response to M-CSF and RANKL but failed to form sealing zones and ruffled borders, displayed disorganized F-actin, and showed impaired secretion of acid and proteases. Mechanistically, LRRK1 regulates multiple components of the osteoclast cytoskeletal and vesicular machinery. LRRK1 modulated c-Src activation by interacting with C-terminal Src kinase (Csk), directly phosphorylated and activated Rac1 and Cdc42, and promoted downstream p21 protein-activated kinase-1 (PAK1) autophosphorylation (Zeng C et al., 2016). LRRK1 also regulated phosphorylation of L-plastin, an actin-bundling protein required for sealing zone assembly, and controled lysosomal distribution and ruffled border formation (Si et al., 2018).

#### 4.4.2 Human disease: osteosclerotic metaphyseal dysplasia

Loss-of-function mutations in LRRK1 caused osteosclerotic metaphyseal dysplasia (OSMD), characterized by undermodeled metaphyses, sandwich vertebrae, and osteoclast-rich osteopetrosis (Howaldt et al., 2020; Iida et al., 2016). Osteoclasts in the patients were large but dysfunctional, exhibiting reduced L-plastin phosphorylation and impaired lysosomal trafficking (Howaldt et al., 2020). Phosphoproteomic analyses revealed altered phosphorylation of VPS35, SNX2/3, VTA1, CTSA, and CFL1, highlighting LRRK1’s broad role in vesicle sorting and cytoskeletal regulation in osteoclasts (Xing et al., 2025).

## 5 Therapeutic target and pharmacology

Several targets involved in osteoclast fusion and polarization are being identified through various pieces of evidence, which hold the potential to become new therapeutic strategies for osteoporosis. The compounds presented here have shown inhibitory effects of bone resorption at both the cellular or animal model levels. Further research is needed to determine whether these compounds are effective and safe in the treatment of human osteoporosis.

### 5.1 Signaling pathways of osteoclast fusion

#### 5.1.1 Targeting DC-STAMP

The small-molecule antagonist of DC-STAMP-tail domain, E8431, selectively disrupted DC-STAMP–RAP1B signaling, preventing preOC fusion while simultaneously promoting anabolic PDGF-BB secretion, thereby protecting against OVX-induced bone loss (Zhang Z. et al., 2025). Administration of E8431 to OVX mice for 6 weeks did not result in pathological changes in the heart, lungs, liver, spleen, or kidneys. However, confirming the safety of long-term administration in humans remains a challenge for the future.

#### 5.1.2 Targeting Siglec-15

Siglec 15 signaling is involved in both the recognition of fusion targets (sialylated TLR2) and the amplification of RANKL signaling (DAP12/Syk, PI3K/Akt, ERK) (Dou et al., 2022; Hiruma et al., 2013; Ishida-Kitagawa et al., 2012; Kameda et al., 2013; Muneyama et al., 2023). Pharmacological strategies include neutralizing antibodies (preclinical), genetic knockdown, and transcriptional suppression (see Cajanin, see 5.1.3). Given mild osteopetrosis in KO mice, Siglec-15 targeting may offer disease-selective antiresorptive effects with lower risk of over suppression of baseline remodeling (Hiruma et al., 2013; Kameda et al., 2013). Our group developed a rat monoclonal anti-Siglec-15 antibody (32A1) and its humanized version (DS-1501a) and explored their therapeutic potential (Tsuda et al., 2022). We showed that 32A1 suppressed osteoclast multinucleation and bone resorption in cultures of mouse bone marrow cells and human osteoclast precursor cells. A single administration of DS-1501a significantly reduced bone resorption markers with minimal impact on bone formation markers and prevented lumbar vertebral BMD loss in OVX rats. Tsukazaki et al. demonstrated through *in vivo* imaging experiments using a pH-sensing probe that anti-Siglec 15 antibody treatment suppressed the bone resorbing-activity of osteoclasts more than treatment with risedronate. (Tsukazaki et al., 2021). Regarding siglec-15 Ab (NP159) as a potential treatment to prevent bone loss in an acute spinal cord injury (SCI) model, Peng et al. reported that administration of NP159 suppressed bone loss in a rat model of acute SCI (Peng et al., 2023). Notably, in osteogenesis imperfecta (OI) models, the humanized Siglec 15 antibody NP159 markedly decreased fracture incidence and enhanced bone structure and mineral composition without reducing bone mineral density, highlighting its unique mechanism of promoting high-quality bone formation (Seide et al., 2025). While it is unclear whether NP159 can suppress bone loss caused by oophorectomy, these studies suggest that Siglec-15 neutralizing antibodies may be also effective in treating bone loss in conditions other than postmenopausal osteoporosis. Very recently, it has been reported that Siglec-15 expression in Kupffer cells is reduced in patients who experience acute rejection after liver transplantation (Luo et al., 2026). This result suggests that the administration of Siglec-15 neutralizing antibodies to osteoporosis patients scheduled for organ transplantation should be approached with caution.

#### 5.1.3 Inhibitors of fusion-regulating transcription/signaling

Natural compounds such as Cajanin further expand the therapeutic landscape by transcriptionally suppressing critical fusion regulators including Siglec15, DC-STAMP, OC-STAMP, and NFATc1 alongside MAPK and NF-κB signaling, resulting in robust inhibition of osteoclastogenesis and preservation of trabecular bone (Ma et al., 2026). Similarly, aconine attenuated RANKL-induced osteoclast differentiation and bone resorption through blockade of NF-κB and NFATc1 activation and downregulation of osteoclastic effector genes, including DC-STAMP (Zeng X et al., 2016). Ma et al. identified a natural compound, morusinol, that specifically destroyed the combination of MSX2 and PU.1. This compound attenuated OVX-induced bone loss.

### 5.2 Cytoskeletal signaling in osteoclasts

#### 5.2.1 Targeting LRRK1

Structure-based screening identified IN04, a selective LRRK1 kinase inhibitor that binds the ATP pocket and suppresses osteoclast resorption without affecting differentiation of osteoclasts or osteoblast function (Si et al., 2019). IN04-treated osteoclasts phenocopied LRRK1 deficiency, failing to form sealing zones and displaying disorganized F-actin. Selective LRRK1 inhibition may therefore offer an antiresorptive strategy that avoids suppression of bone formation. Whether IN04 exerts bone resorption inhibitory effects *in vivo* remains a subject for future research. Dual LRRK1/LRRK2 inhibitors such as GZD824 exist, but their skeletal effects remain unexplored (Malik et al., 2021).

#### 5.2.2 Targeting Rho-family GTPase pathways

Pharmacological targeting of intracellular regulators of osteoclast cytoskeletal dynamics has emerged as a promising strategy for treating osteolytic disorders. Inhibition of the guanine nucleotide exchange factor Dock5, which is essential for Rac activation and formation of the osteoclast sealing zone, effectively suppresses bone resorption without impairing osteoblast-mediated bone formation. The small-molecule inhibitor C21 directly blocked Dock5 exchange activity, disrupted podosome organization, and protected against bone degradation in multiple murine models of osteoporosis, rheumatoid arthritis, and cancer-induced osteolysis, all without detectable toxicity (Vives et al., 2015). Similarly, inhibition of PKN3, a downstream effector of Wnt5a–Ror2 signaling in mature osteoclasts, attenuates osteoclast function while preserving osteoclast and osteoblast numbers. The PKN3-targeting compound SB202190 reduced actin ring formation, suppressed resorption-pit formation, and prevented OVX-induced bone loss *in vivo*, positioning PKN3 as a compelling therapeutic target for pathological bone resorption (Uehara et al., 2022). These findings, taken together, highlight the therapeutic value of selectively inhibiting the cytoskeleton and signaling mechanisms of osteoclasts. Furthermore, it is suggested that inhibiting small GTPases or regulating small GTPase regulators such as GAP and GEF may be effective strategies for controlling osteoclast-induced bone loss. While both Dock5 and PKN3 are highly expressed in osteoclasts and less expressed in other tissues, it remains unclear how inhibitors of these targets affect tissues other than bone.

## 6 Conclusion

Osteoclast fusion and polarization are increasingly recognized as highly selective therapeutic entry points for osteolytic diseases. Fusion is controlled by transcriptional fusogens such as DC-STAMP, OC-STAMP, Atp6v0d2, and NFATc1, by recognition modules including Siglec-15, CD47–SIRPα, CD9, and integrins, and by membrane-mechanical systems involving phosphatidylserine exposure, annexins, ERM proteins, and BAR-domain regulators. These pathways coordinate the formation of tunneling nanotubes and zipper-like structures that enable precise precursor pairing and fusion pore formation. Pharmacological strategies such as DC-STAMP antagonists (e.g., E8431), and Siglec-15 pathway inhibitors along with multi-node compounds like cajanin illustrate the growing feasibility of developing fusion-selective antiresorptives that preserve or even enhance osteogenic coupling.

In parallel, osteoclast resorptive function is driven by integrin–Src–Pyk2 scaffolding, Rho-family GTPases, and LRRK1-dependent cytoskeletal organization, offering additional opportunities to modulate bone resorption without suppressing bone formation. Targeting these signaling hubs including upstream GAP/GEF regulation holds promise for precisely reducing osteoclast activity while maintaining, or potentially promoting, bone-forming signals.

Collectively, inhibiting osteoclast fusion or polarization represents a compelling therapeutic strategy for osteoporosis and other bone-loss disorders. As a future direction, an optimal osteoporosis treatment program may emerge from combining three complementary approaches: (i) conventional agents such as bisphosphonates and denosumab to reduce osteoclast number, (ii) fusion-inhibitory strategies to moderately suppress resorption while enhancing Type H vasculature, and (iii) polarization-targeting strategies that dampen bone resorption yet preserve coupling-factor–mediated bone formation. Such tailored combinations may ultimately provide patients with more effective and physiologically balanced osteoporosis care.
